# Adaptation of clinical bacteriology techniques for remote polar research

**DOI:** 10.1128/aem.02147-24

**Published:** 2025-01-16

**Authors:** Scott J. C. Pallett, Bill K. Kwok, Stephanie M. Y. Wong, Luke S. P. Moore

**Affiliations:** 1 Centre of Defence Pathology, Royal Centre for Defence Medicine, Queen Elizabeth Hospital 156807 , Birmingham, United Kingdom; 2 Clinical Infection Department, Chelsea and Westminster Hospital NHS Foundation Trust 9762 , London, United Kingdom; 3 Royal Geographical Society 11467 , London, United Kingdom; 4 National Institute for Health Research Health Protection Research Unit in Healthcare Associated Infections and Antimicrobial Resistance, Imperial College London - Hammersmith Campus 156647 , London, United Kingdom; Colorado School of Mines, Golden, Colorado, USA

**Keywords:** remote diagnostics, arctic, microbiology

## Abstract

Remote polar regions offer unique opportunities and significant challenges for antimicrobial resistance research in a near-pristine environment. While core microbiology techniques continue to have an important role in supporting environmental research, the severe cold climate presents considerable challenges to laboratory research. We explore adaptations required for core bacteriology investigations in polar regions on an unsupported remote expedition c. 600 km north of the Arctic Circle utilizing the National Collection of Type Culture bacterial strains. Methods of culture, microscopy, biochemical and phenotypic testing, vortex, and centrifuge techniques are explored. Across −21.5 to −41.0°C, culture was satisfactorily enabled using a solar-powered USB incubator and an electricity-free water-bath option utilizing white gas for a variety of standard culture media. Microscopy and biochemical tests supported organism identification. Phenotypic testing for carbapenemase-producing genes using lateral flow devices showed good performance without modification (Carba-5, 20/20 carbapenemase-producing organism tests, 100% sensitivity; 100/100 negative targets, 100% specificity). The modified centrifuge was enabled with a 3D printed disk and Dremel drill and microbial DNA extraction (ZymoBIOMICS) kits were able to extract DNA of suitable quality for analysis. With suitable adaptations, conducting core microbiology techniques (with potential relevance for more advanced techniques) is possible in the remote extreme cold environment.

**IMPORTANCE:**

Antimicrobial resistance (AMR) represents one of the key global public health threats currently facing humanity. The recent UN High-Level Meeting on AMR highlighted the need for greater knowledge generation on its environmental aspects while also considering the potential adverse effects of climate change. The polar regions of the world offer a unique opportunity for AMR research in a near-pristine environment while also holding the potential for novel resistance mechanisms and/or antimicrobial peptide discovery within melting permafrost or glacial ice. Despite considerable technological advances in microbiology, operating in severe cold environments continues to present significant operational challenges. Our report here offers a basis for adaptations to enable both environmental and clinical antimicrobial resistance, microbiome, and discovery research for operating in the harshest of remote environments.

## INTRODUCTION

When Captain RF Scott RN returned in 1903 from the historic *Discovery* expedition to Antarctica, it ushered in over a century of British polar scientific endeavor that continues today (1, 2). While Captain Sir Alexander Fleming would not make his discovery of penicillin until 1928, many early expeditions coincided with the “golden” age of microbiology. They were directly advised by renowned pathologists of the time (2). Despite this, reporting of human bacteriology was scarce, being considerably limited by the challenges of conducting microbiology (and, in particular, bacterial culture) in this extreme environment (2, 3).

In the twenty-first century, there remains considerable interest in polar microbiology and environmental research. Antimicrobial resistance is recognized by the World Health Organization as a key public global health threat and a priority area for research, while the UK Fleming Fund has recently highlighted concerns for thawing permafrost and the risk of emerging pathogens, including novel antimicrobial resistance genes, being released into the environment (4). Polar regions today, largely uninhabited by humans, offer a unique environment to understand and model interactions between climate change and antimicrobial resistance and may present opportunities for novel antimicrobial mechanism discovery (5). Recent research has highlighted the potential for AMR gene discovery within ice polar regions, including major carbapenamase families (6–8). Persistent human activity at inhabited polar research stations, however, means these occupied regions are likely to be limited in their capacity to offer an optimal pristine environment (9). Future research may also benefit from more remote, small-team expeditions, similar to the small-footprint teams of the past.

Over the last 100 years, the practice of microbiology has changed significantly in the availability of techniques (e.g., molecular assays) and technology (e.g., solar and battery power) yet core techniques continue to be practiced on a daily basis as integral elements of both clinical and academic bacteriology, including in the field (10). Where modifications are made to enable techniques in the extreme cold, it is important to ensure quality through concordance with clear standards such as the UK Standards of Microbiology (UKSMI) (11). We have, therefore, undertaken a remote, exploratory unsupported polar expedition and report on modern challenges of conducting core traditional and modern microbiology techniques for preliminary identification of medically important organisms in extreme environments (11).

## MATERIALS AND METHODS

**Fig 1:**
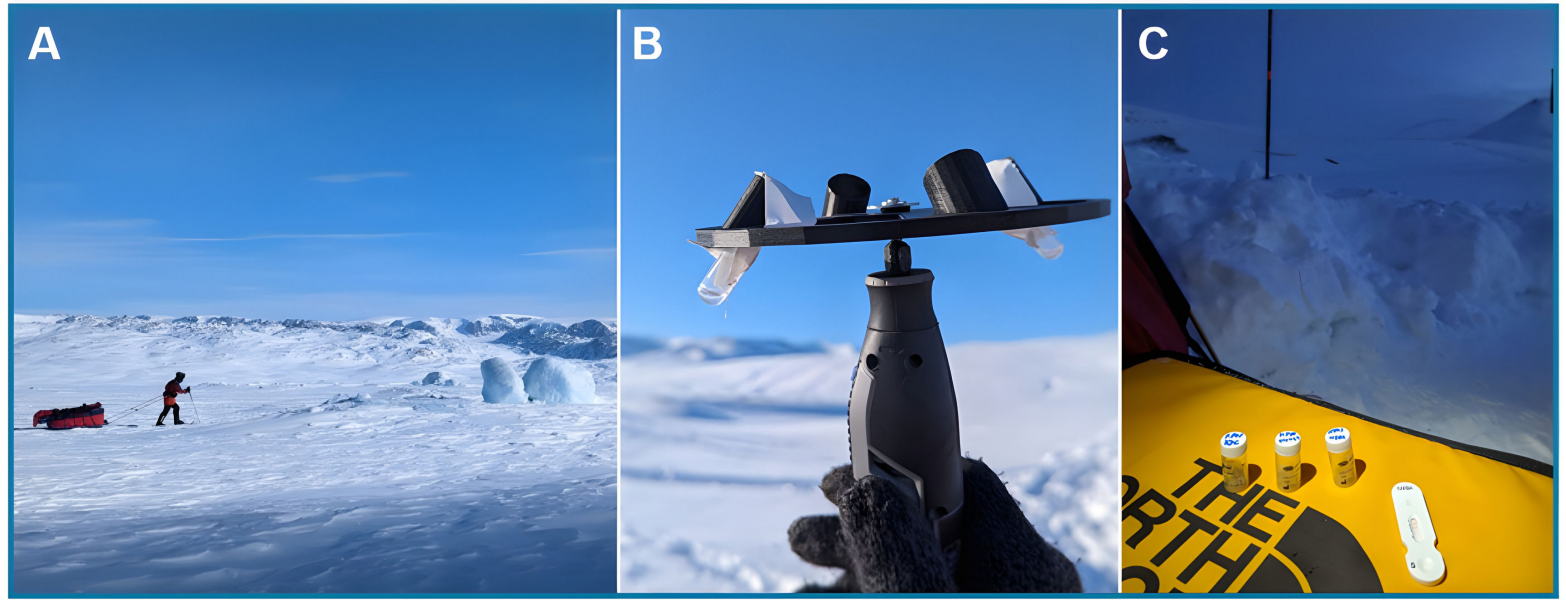
Assessing challenges of core bacteriology techniques in Arctic conditions. (**A**) Crossing sea ice on skis with modified laboratory equipment pulled behind in a pulk at −41.0°C. (**B**) A 3D-printed four-column centrifuge plate mounted on a wireless Dremel drill. (**C**) Lateral flow testing (Carba-5) of *K. pneumoniae* samples from solid nutrient agar media at −27.5°C.

A remote polar expedition was conducted to explore the performance and innovations required to enable an array of core microbiology skills in a remote, exposed environment of −20°C and below. The expeditionary work was unsupported with necessary laboratory and survival equipment carried between two pulks and pulled on skis through uninhabited regions of Jameson Land and Scoresby Land, Greenland in March 2024 (Fig. 1), powered using an Anker 20,000 mAh power bank.

### Culture, microscopy, and biochemical techniques

Traditional bacteriology techniques were assessed including liquid and solid agar media culture, microscopy, and biochemical tests used to assist in organism identification [coagulase, catalase, spot indole, oxidase, and use of analytical profile index (API, bioMerieux, USA) strips] as per UKSMI recommendations (11). National Collection of Type Culture samples were utilized for testing for example, medically important Gram-positive (NCTC 6571 *Staphylococcus aureus*) and Gram-negative (NCTC 9633 *Klebsiella pneumoniae*) organisms. Ceramic beads (Pro-Lab Diagnostics, UK) were used for the transport of samples. Culture was compared using (i) a small USB-charged incubator powered by a 20,000 mAh power bank, recharged using a 120-W solar panel, (ii) a water bath heated using a white gas stove and maintained in a 750 mL insulated thermos, and (iii) a 20 L drybag and two cycled USB-rechargeable hand warmers. Culture temperatures were monitored using a USB-enabled lithium battery-powered thermometer, accurate to ±0.1°C (lower range −40°C) with recording of temperature at 2 s intervals. Culture media tested included commercial Columbia blood agar (CBA) and chocolate agar plates, non-selective solid nutrient agar slopes (containing 0.5% peptone, 0.3% beef extract prepared and prepared onsite), and liquid nutrient broth media (without agar, prepared onsite). These media were selected for the initial assessment due to their commercial availability. The incubation methods were assessed out to 10 h at a time. Colonies were inspected for morphology and then collected for further testing at 4 h incubation (35–37°C). Bacterial pathogens were characterized using a USB-charged light microscope (following Gram stain) and biochemical tests as per UKSMI (11). Additional test performance was verified using NCTC strains (indole: NCTC 10418 *Escherichia coli* and oxidase: NCTC 10662 *Pseudomonas aeruginosa*). Nitrile gloves were used when handling samples, but these required switching to a warm pair of gloves for tasks in-between to reduce any issues with manual dexterity. Inside the tent, wearing nitrile gloves for up to 10 min at a time during microscopy was achievable.

### Phenotypic testing for AMR genes

Phenotypic tests for antimicrobial resistance genes were screened as an example for lateral flow devices (LFTs; Carba-5, NG-Biotech) using five tests each for *K. pneumoniae* NCTC strains 9633 (no resistance mechanism), 13422 (OXA-48 producing), 13443 (NDM-1 producing), 13438 (KPC producing), and a known NDM/OXA-48 co-producing *K. pneumoniae* (confirmed using molecular testing via the national reference laboratory) organism from an infected wound sample. Resistant samples were extracted/inactivated prior to prevent any risks of contamination, transporting/travelling, in keeping with the aim to test the device, not the organism.

### Isolate preparation to enable modern sequencing techniques

Vortex was tested using a USB-powered mini paint mixer and a centrifuge of samples was conducted using a 3D printed disk with space for 4 × 15 mL Falcon tubes and powered by a wireless Dremel drill with ranged settings from 10,000 to 33,000 rpm (Fig. 1). Samples (including bacterial culture, soil, and fungi) underwent nucleic acid extraction using ZymoBIOMICS DNA miniprep kits as per manufacturer instructions. Extracted DNA quality control was subsequently undertaken by Nanodrop (cut-off >10 ng/mL), Qubit (cut-off >1 ng/mL), and gel electrophoresis (UK, Aligent Technologies TapeStation 4200) suitable for testing in remote regions or to make suitable for safe transport back to a standard research facility for further testing. Results in the field were recorded electronically using a Panasonic Toughbook CF-19, i5 Rugged laptop, running Windows 10, powered by an Anker 20,000 mAh power bank and recharged using a 120-W foldable solar panel.

## RESULTS

Remote testing of techniques was conducted across 250 km of unsupported polar travel. Each step of testing was repeated on four separate days at recorded temperatures of –21.5,–27.5, −33.0, and −41.0°C.

### Culture and microscopy

Despite careful storage of agar plates, some degree of freezing was experienced across all days, affecting CBA more than chocolate agar plates. After gentle thawing, a small amount of liquid media required discarding (this was done to prevent a top layer of freezing agar). The USB-incubator method was adequately powered using an Anker 20,000 mAh power bank. To reach a consistent 35–37°C, it required storage in a 2 cm thick fitted polystyrene box, with a removable lid to allow easy access to plates and wrapping in a layer of WoolCool, leaving the front exposed before placing inside a black, water/wind-proof dry-bag. Using this method, satisfactory temperatures were reached within 20 min and maintained as required, assessed out to 10 h. Water temperature in the water bath reached 37°C within 15 min and was maintained for 2 h 7 min (−41.0°C real-feel) to 2 h 52 min (−21.5°C real-feel), at which point replacement of hot water was required to maintain suitable temperatures. The culture was most successful with the use of nutrient agar slopes, then liquid nutrient agar, and then pre-poured agar plates (Table 1). The hand-warmer incubator design was able to reach suitable temperatures when combined with a WoolCool layer but was unable to maintain a stable 35–37°C beyond 55 min, no colonies were seen and, therefore, no further testing was conducted.

**TABLE 1:** Culture performance in polar conditions using modified USB-incubator, thermos water-bath, and handwarmer approaches^*a*^

Test	Growth		Gram stain		Biochemical tests					
Sample	*S. aureus*	*K. pneumoniae*	*S. aureus*	*K. pneumoniae*	Sa coagulase	Sa catalase	Kp catalase	Kp indole	Kp oxidase	API 20E
Culture method										
Incubator: CBA	+	++	GPC clus	GNR	+	+	+	–	–	Kp
Incubator: chocolate	+	++	GPC clus	GNR	+	+	+	–	–	Kp
Incubator: solid NA	++	+++	GPC clus	GNR	+	+	+	–	–	Kp
Water bath: liquid NB	+	++	GPC clus	GNR	+	+	+	–	–	Kp
Water bath: solid NA	++	++	GPC clus	GNR	+	+	+	–	–	Kp
Hand warmer (all)	NG	NG	NG	NG	/	/	/	/	/	/

^*a*^ Sa = NCTC 6571 *Staphylococcus aureus*, Kp = NCTC 9633 *Klebsiella pneumoniae*, GNR = Gram-negative rods, GPC clus = Gram-positive occi in clusters, NA = nutrient agar, NB = nutrient broth, CBA = Columbia blood agar, API = analytical profile index. NG = no growth, / = not applicable. For growth columns, the plus symbol (+) was used to indicate poor growth (+), moderate growth (++) or good growth (+++). For biochemical tests, the plus symbol (+) indicates a positive test result and a minus symbol (-) indicates a negative test result. Results were consistent as presented across tests conducted at real feel temperatures of –21.5,–27.5, −33.0, and −41.0°C. Incubator tests were conducted using a USB-powered incubator, encased in 2 cm polystyrene, and wrapped in WoolCool before being placed inside a black dry bag. Water-bath testing was conducted using a 750 mL thermos with water heated by white gas. Temperatures were maintained between 35 and 37°C and microscopy/biochemical testing was conducted at 4 h. Hand-warmer incubation was unable to maintain adequate temperatures to enable growth at 55 min CBA, chocolate and solid nutrient agars were removed and no growth was identifiable.

**Fig 2:**
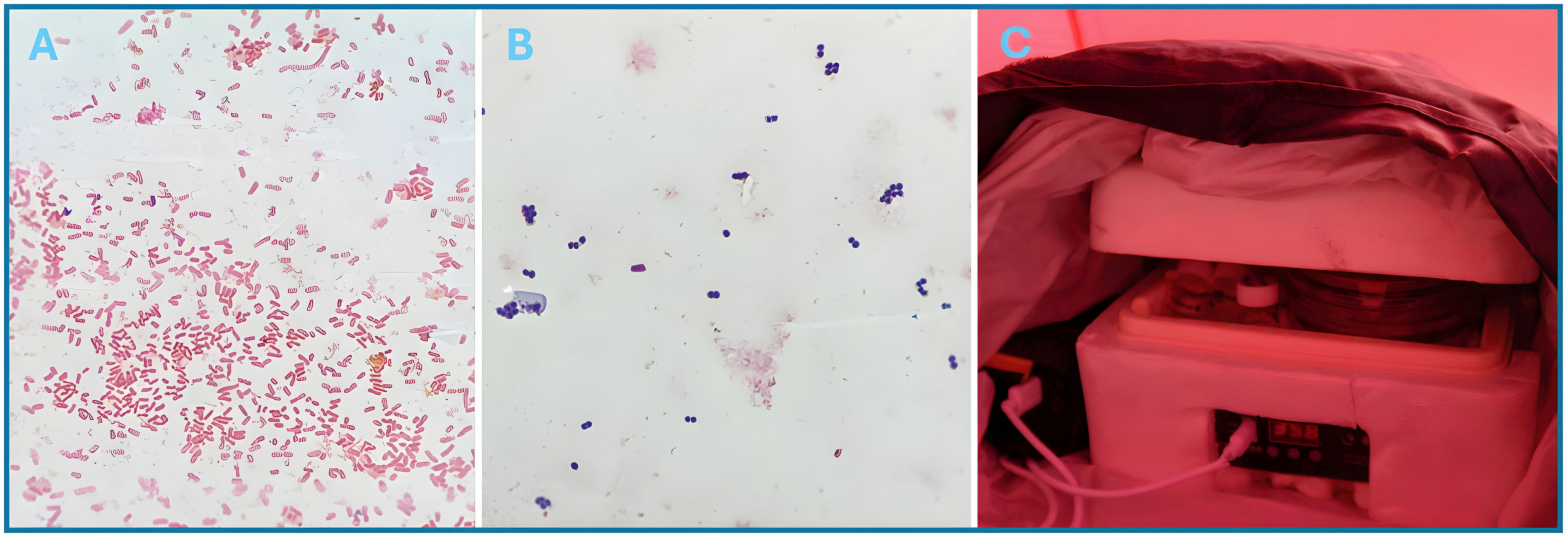
Microscopy of organisms grown on solid nutrient agar using a USB incubator in polar conditions. Gram stains were conducted on colonies collected following culture at 35–37°C for 4 h using a USB-powered incubator encased in 2 cm polystyrene and wrapped in WoolCool before being placed inside a black dry bag. The real-feel temperature at the time was −33°C. Microscopy was conducted at 1000× magnification. (**A**) Gram-negative rods on Gram staining of NCTC 9633 *Klebsiella pneumoniae* colonies. (**B**) Gram-positive cocci in clusters on Gram staining of NCTC 6571 *Staphylococcus aureus.* (**C**) Set up for USB incubator, powered by a 20,000 mAh power bank (left side). To maintain temperature, the incubator was encased in 2 cm polystyrene, wrapped in WoolCool, and placed inside a black dry bag.

All reagents for phenotypic and biochemical tests and Gram stain froze. Reagents were gently thawed as required by (i) using a specialized thermos, modified by replacing the lid with a Fresnel lens or where not appropriate (poor direct sunlight) by (ii) placing them in a dry bag with a USB-powered hand warmer and carried in a chest pocket. Organism biochemical properties were confirmed for each culture type at each test occasion (Table 1). The heat-fix step of microscopy was satisfactorily enabled by (i) intermittent exposure to a handheld lighter flame and (ii) placing the slide atop a cooling stove pot previously heated by white gas. Microscopy needed to be conducted in the vicinity (i.e., <30 cm) of the white gas stove to prevent immediate freezing of the sample on the slide. A total of 16 microscopy slides were made (4× NCTC *S. aureus*, Gram-positive cocci in clusters) and (4× NCTC *K. pneumoniae*; Gram-negative rods) (Fig. 2).

### Phenotypic antimicrobial resistance testing

25 Carba-5 LFTs were performed from culture (Fig. 1C). All tests were performed adequately with the detection of control lines. Sensitivity against known resistance phenotypes was 100.0% (20/20). Specificity testing was calculated for a total of 100 negative gene targets with no false positive test results seen (100/100, 100%).

### Assessment of adapted kit to enable modern sequencing techniques

The vortex worked with intermittent battery success, requiring 5 min warming prior to use (held within jacket). The modified Dremel drill had no issues enabling the centrifuge at ranges of 10,000–33,000 rpm. DNA extraction provided suitable quality extraction for bacteria and soil (Nanodrop 46.1 ng/mL, 17.6 ng/mL, respectively) without bead beating.

## DISCUSSION

While the extreme cold environment remains challenging for undertaking core bacteriology investigations, the use of modern laboratory techniques is suitably reproducible with simple innovations.

Difficulty in conducting bacterial culture, particularly in maintaining stable temperatures for sufficient durations and in cracking of solid agar, was highlighted on the Australasian expedition of 1911 (3). Adaptations to enable remote incubation have been described following use in Canada (10), but these still require a reasonable amount of equipment, impacting the weight burden of transporting mobile, remote laboratories on skis. Our USB-incubator method was able to overcome these challenges but is also reliant on clear sunlight (or carrying of a small generator) and so is less reliable during periods of long nights. While we were able to maintain powerbanks using solar panels, we were limited by overcast days, and during temperatures below −20°C, we noted there was a risk of charge leakage in the opposite direction unless the powerbanks were carried on the body. Water baths, using nutrient agar media, provided a far more reliable option. Eppendorf or Falcon tubes for creating slopes or liquid agar media are lightweight and require considerably less space or care than pre-poured agar plates while avoiding measures required to try and protect them from freeze-thaw. Suitable temperatures were found to be easily maintained in a compact 750 mL water-bath incubator using white gas, carried as a necessity in this environment, and snow which was readily available.

Microscopy of cultures acquired via these two methods were both comparable and suitable for supporting bacterial identification (Fig. 1). As technology continues to develop, smaller digital microscopes may reduce the weight burden further although these would need to be powered by lithium batteries to enable use in colder temperatures. Together with benchside tests, presumptive identification of *Staphylococcus aureus,* a key potential skin and soft tissue pathogen in this environment, Enterobacterales and *Pseudomonas* spp. were possible. API, while possible, required the larger powered incubator and so presumptive diagnosis of Gram-negative organisms, to genus rather than species level, may have to rely on a small collection of individual bench-side tests.

Adequate performance of LFTs enables scope for additional phenotypic screening in cold environments where key targets of concern are suspected or known. The discovery of novel enzymes within wastewater-contaminated ice on Svalbard from major β-lactamase families could enable LFTs to be used as a screening tool for the recovery of samples suitable for sequencing (7). While other individual kits would need to be assessed separately, LFTs are lightweight simple, and cost-effective tests that could offer some diagnostic capability across the identification of bacterial or viral infections or immunological responses depending on the research question. This is reassuring in scenarios beyond polar research including for screening of antimicrobial resistance genes in areas of high AMR burden. The ongoing conflict in Ukraine, for example, has been shown to have high rates of AMR complicating wound infection with sub-zero winter temperatures in remote regions (12). New adaptations to these assays, allowing for testing directly from the sample (13), could potentially provide a realistic early screening tool in remote temporary medical facilities with limited laboratory infrastructure. The performance of adapted vortex and centrifuge equipment are integral steps to considering remote molecular testing including polymerase chain reaction, loop-mediated isothermal amplification, or sequencing assays. DNA extraction was likewise shown to be possible, raising the possibility of remote sequencing (e.g., via a nanopore MinION device), or alternatively an option to extract nucleic acids for safer transport. Options to reduce preparation times may be required to overcome the risks of spending extended periods extracting nucleic acids (requiring high finger dexterity) at −20°C and below.

The study was limited by testing of example organisms/assays and further assessment would be required to understand the performance of additional tests (e.g., LFTs from other manufacturers) or more fastidious organisms. The study does, however, provide real-world confirmation of the feasibility of culture, for both solar-powered and electricity-free options, and the adaptability of core techniques in extreme cold scenarios that are necessary for more advanced (e.g., molecular or sequencing) techniques.

### Summary

Despite significant advances in technology, core microbiology skills remain relevant in supporting bacterial diagnosis and discovery. With suitable adaptations, conducting of core microbiology techniques, cognisant of UKSMI or equivalent requirements, is possible in the remote extreme cold environment.
